# Ginkgolide C Alleviates Myocardial Ischemia/Reperfusion-Induced Inflammatory Injury via Inhibition of CD40-NF-κB Pathway

**DOI:** 10.3389/fphar.2018.00109

**Published:** 2018-02-21

**Authors:** Rui Zhang, Dan Han, Zhenyu Li, Chengwu Shen, Yahui Zhang, Jun Li, Genquan Yan, Shasha Li, Bo Hu, Jiangbing Li, Ping Liu

**Affiliations:** ^1^Department of Pharmacy, Shandong Provincial Hospital Affiliated to Shandong University, Jinan, China; ^2^Department of Pharmacy, Nanjing Drum Tower Hospital, The Affiliated Hospital of Nanjing University Medical School, Nanjing, China; ^3^Minimally Invasive Urology Center, Shandong Provincial Hospital Affiliated to Shandong University, Jinan, China; ^4^Department of Cardiology, Shandong Provincial Hospital Affiliated to Shandong University, Jinan, China

**Keywords:** ginkgolide C, myocardial ischemia/reperfusion injury, inflammation, CD40, NF-κB

## Abstract

Increasing evidence shows that inflammation plays a vital role in the occurrence and development of ischemia/reperfusion (I/R). Suppression of excessive inflammation can ameliorate impaired cardiac function, which shows therapeutic potential for clinical treatment of myocardial ischemia/reperfusion (MI/R) diseases. In this study, we investigated whether Ginkgolide C (GC), a potent anti-inflammatory flavone, extenuated MI/R injury through inhibition of inflammation. *In vivo*, rats with the occlusion of the left anterior descending (LAD) coronary artery were applied to mimic MI/R injury. *In vitro*, primary cultured neonatal ventricular myocytes exposed to hypoxia/reoxygenation (H/R) were applied to further discuss the anti-H/R injury property of GC. The results revealed that GC significantly improved the symptoms of MI/R injury, as evidenced by reducing infarct size, preventing myofibrillar degeneration and reversing the mitochondria dysfunction. Moreover, histological analysis and Myeloperoxidase (MPO) activity measurement showed that GC remarkably suppressed Polymorphonuclears (PMNs) infiltration and ameliorated the histopathological damage. Furthermore, GC pretreatment was shown to improve H/R-induced ventricular myocytes viability and enhance tolerance of inflammatory insult, as evidenced by suppressing expression of CD40, translocation of NF-κB p65 subunit, phosphorylation of IκB-α, as well as the activity of IKK-β. In addition, downstream inflammatory cytokines modulated by NF-κB signaling were effectively down-regulated both *in vivo* and *in vitro*, as determined by immunohistochemistry and ELISA. In conclusion, these results indicate that GC possesses a beneficial effect against MI/R injury via inflammation inhibition that may involve suppression of CD40-NF-κB signal pathway and downstream inflammatory cytokines expression, which may offer an alternative medication for MI/R diseases.

## Introduction

Nowadays, cardiovascular disease becomes one of the leading cause of disability and death worldwide, and one of the most popular and dangerous cardiovascular diseases is myocardial ischemia/reperfusion (MI/R) injury (Anzell et al., 2017; de Waha et al., 2017). While prompt reperfusion is favorable for myocardial salvage, ischemia/reperfusion (I/R) injury which involved a strong inflammatory response may often present itself as an adverse consequence (Liebert et al., 2017). According to the recent researches, an inflammatory response severely ignites the whole process of MI/R injury by accelerating generation of inflammatory factors and by boosting inflammatory eruption simultaneously (Montecucco et al., 2017). A disturbed inflammatory stimuli is associated with poor prognosis and increased tissue damage, may aggravate the degree of I/R injury and it may seriously delay the recovery procedures (Pantazi et al., 2016; Yarijani et al., 2017). Therefore, restoration of cardiac dysfunction through modulation of inflammatory pathways is a favorable therapeutic strategy against MI/R diseases superimposed on other cardiovascular disease risk factors.

CD40, one member of tumor necrosis factor receptor (TNFR) family, serves as a transmembrane type I receptor (Clark, 2014). Except for immune cells, CD40 has been similarly found on endothelial cells, myocytes, epithelial cells, fibroblasts, and monocytes (Senhaji et al., 2015). Tumor necrosis factor (TNF)-α, Interleukin (IL)-1, Interferon (IFN)-γ, CD40 ligand (CD40L) and others inflammatory factors can regulate the expression of CD40. Then, the abundant expression of CD40 in the cytoplasmic domain will tend to TNF receptor-associated factors (TRAFs), which consequently activates different signal pathways, such as nuclear factor-κB (NF-κB), phosphoinositide 3-kinase (PI3K) and MAPKs, in different stages of MI/R injury process (Jansen et al., 2016). Researches for near years have indicated that CD40 was closely related to the occurrence and development of many diseases, including lung ailments, inflammatory kidney disease, diabetes, coronary artery disease and arteriosclerosis disease (Aloui et al., 2014; Mansouri et al., 2016; Michel et al., 2017). However, few studies investigated the role of CD40 in the whole inflammatory process induced by MI/R injury.

Increasing evidence suggested that NF-κB activation elevated in the MI/R-related infarct area, inflammation was suppressed when NF-κB activation was inhibited, and cardiac preservation was provided (Zhang et al., 2017). IκB-α, which binds to NF-κB p65/p50 heterodimer in the cytoplasm under the rest state, serves as an inhibitor of NF-κB. Subsequently, IκB kinase β (IKK-β) activation induces the phosphorylation and degradation of IκB-α resulting in the translocation of NF-κB from cytoplasm to nucleus, which will enhance synthesis of various inflammatory cytokines, such as TNF-α, IL-1β, IL-6, vascular cell adhesion molecule-1 (VCAM-1), intracellular adhesion molecule-1 (ICAM-1) and inducible nitric oxide synthase (iNOS), that act directly or indirectly to depress cardiac function (Durand and Baldwin, 2017; Nennig and Schank, 2017). However, many studies have reported that aspirin possesses the strong anti-inflammatory properties through inhibiting the transcription factor such as NF-κB (He et al., 2012). Moreover, neutrophils infiltration into the post-ischemic myocardium has previously been related to the pathogenesis of I/R injury. Reperfusion accelerates the recruitment of neutrophils into the ischemic myocardium (Sivalingam et al., 2017). Thus, suppressing neutrophils infiltration and NF-κB pathway activation can diminish MI/R damage and possibly improve myocardial function. From this evidence, it was reasonable to speculate that NF-κB signal pathway played a key role throughout the whole course of MI/R injury. Therefore, potent new agents for treatment of MI/R injury via anti-inflammatory effect become an imperative requirement.

**FIGURE 1:**
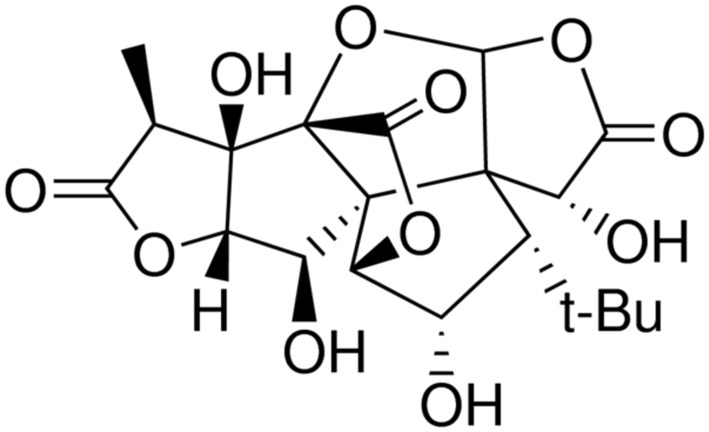
Chemical structure of GC.

Ginkgolide C (GC, **Figure 1**), isolated from *Ginkgo biloba* leaves, is a flavone with multiple biological functions and has a long application history in clinical therapy in Asia (Huang et al., 2014). It was reported that GC exerted protection of ischemic diseases, inhibition of platelet aggregation, prevention of thrombus, anti-inflammation, and anti-allergy (Lau et al., 2013; Liou et al., 2015). However, up to now, there is little research on the relations between GC and MI/R injury, and the exact mechanism of its anti-inflammatory protective effects is still obscure to us.

Thus, taking into account activation of inflammatory response during MI/R injury, we have not only investigated the protective effect of GC both *in vivo* and *in vitro*, but also made great efforts to clarify the potential mechanism. Considering the supposed signal pathway of GC, aspirin which represents the prototype of anti-inflammatory and MI/R protective effects was selected as the positive control in this study. Left anterior descending (LAD)-occlusion-induced myocardial infarction rat model was established to imitate MI/R *in vivo*. Moreover, ventricular myocytes treatment with hypoxia/reoxygenation (H/R) were used to mimic I/R injury *in vitro*. To further explore the mechanism, we also explored the role of CD40-NF-κB signal pathway in the protection of ventricular myocytes treated with H/R.

## Materials and Methods

### Materials and Reagents

GC (PubChem CID: 161120), Aspirin (PubChem CID: 2244), 2, 3, 5-Triphenyltetrazolium chloride (TTC), 3-(4, 5-dimethylthiazol-2-yl)-2, 5-diphenyltetrazolium bromide (MTT) and chloral hydrate were bought from Sigma (St. Louis, MO, United States). DMEM medium (high glucose) and newborn calf serum were products of Gibco (Grand Island, NY, United States). Trypsin was purchased from Amresco (Solon, OH, United States). TNF-α, IL-1β and IL-6 ELISA kits were bought from Sigma (St. Louis, MO, United States). Anti-ICAM-1, anti-VCAM-1, anti-iNOS, anti-CD40, anti-NF-κB p65, anti-p-IκB-α, anti-IKK-β, anti-β-actin, anti-Histone, goat anti-rabbit and anti-mouse IgG antibodies were purchased from Santa Cruz (Santa Cruz, CA, United States). Nuclear-Cytosol Extraction Kit and ECL plus kit was purchased from Jiancheng (Nanjing Jiancheng Bioengineering Inc., Nanjing, China)

### Animals

Adult male Sprague-Dawley rats (250 ± 20 g) were provided by Experimental Animal Center of Shandong University. Rats were raised in a controlled environment (temperature 18–22°C, humidity 40–70%) with a 12 h light-dark cycle and allowed to eat and drink freely until the experiment. All the experiments were approved by the ethics committee of the Shandong University (Permission No. 2013-092).

### *In Vivo* I/R Procedure to Induce MI/R Injury in Rats

**FIGURE 2:**
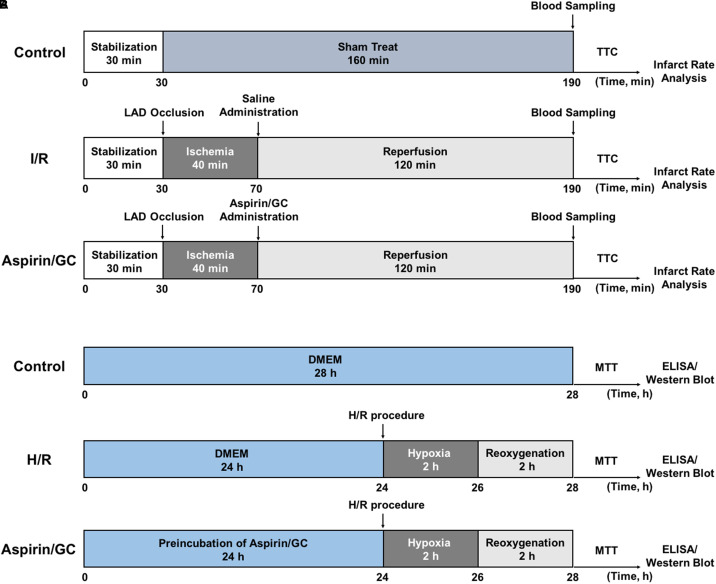
*In vivo* I/R procedure **(A)** and *in vitro* H/R procedure **(B)**.

MI/R surgery was precisely implemented according to the procedure in **Figure 2A**. The rats were anesthetized with 3% sodium pentobarbital (40 mg/kg, i.p.) and mechanically ventilated the lung with a rodent respirator. Holter monitoring electrocardiogram was continuously used to monitor the changes of S-T segment in order to estimate the success of surgery. After a left thoracotomy, a suture tied with a plastic tube was twined round the LAD coronary artery to form a snare for reversible LAD occlusion. According to procedure, transient regional myocardial ischemia for 40 min was realized by straining the suture, and reperfusion for 120 min was initiated by releasing the suture and removing the tension. The blood samples were collected before the rats were sacrificed.

Rats were randomly divided into 6 groups as follows (*n* = 8 per group): Control group, non-I/R rats received saline; I/R group, I/R rats received saline; Aspirin group, I/R rats received 16 mg/kg of Aspirin; 8, 16, 32 mg/kg GC group, I/R rats received 8, 16, 32 mg/kg of GC. Saline, Aspirin and GC were administered intraperitoneally for 7 days before I/R procedure.

### Measurement of Infarct Size

Infarct size was assessed by TTC staining technique in accordance with a previous study report (Panda et al., 2017). After the completion of the experimental protocols, the hearts were removed. The left ventricle area was sectioned into five 2–3 mm-thick slices from the apex to the base. The third slice was incubated in pH 7.4, 2% TTC at 37°C for 15 min. Viable tissue was stained to dark red while the infarct area remained pale. Then, the area of the infarcted tissues was demarcated and measured digitally using Image-Pro Plus software (version 6.0, Media Cybernetic, United States) by computerized planimetry. Infarct size was calculated as the ratio of infarcted myocardium to the risk region × 100%.

### Transmission Electron Microscope (TEM) Observation

The third heart slice was fixed in 3.0% glutaraldehyde buffered fixative (pH 7.2) for 2–3 days. After being rinsed in PBS, the specimens were embedded in Polybed 812. Then, 90 nm-thick specimens were made and observed under electron microscope (JEM-2000EX).

### Histopathological Analysis and Qualitative Observation of PMNs Infiltration

The third heart slice was stained with hematoxylin and eosin (HE) after fixation. The degree of histopathological damage was assessed with pathological scores according to the criteria reported by the previous study (Jiang et al., 2012). Scores from 0 to 5 represented no damage, mild damage, moderate damage, severe damage and highly severe damage, respectively. The total number of PMNs was also recorded simultaneously. The degree of PMNs infiltration of the area at risk was determined in three random high-power fields (HPF).

### Immunohistochemistry

The expressions of ICAM-1, VCAM-1 and iNOS were examined by immunohistochemistry. In brief, the third frozen heart slice incubated with anti-ICAM-1, anti-VCAM-1, and anti-iNOS antibodies overnight at 4°C after being blocked by 10% normal serum. Then, anti-rabbit IgG was applied to incubate the heart slices combined with primary antibody for 30 min. Consequently, immunohisochemical staining protocol was applied to heart slice in accordance with the instructions. Images were obtained using fluorescence microscope. The optical density of positive staining area was quantified by Image-Pro Plus software (version 6.0, Media Cybernetic, United States) and the results were expressed as mean optical density mean ± SD.

### Quantitative Determination of MPO Activity

The homogenized tissue samples were sonicated to release the MPO from tissue into the supernatant. Then, *o*-dianisidine hydrochloride and hydrogen peroxide were added, after that, MPO activity was tested at 460 nm according to spectrophotometer method during a period of 3 min. The number of PMNs was counted in three random high power fields.

### *In Vitro* H/R Procedure to Induce H/R Injury in Ventricular Myocytes

Ventricular myocytes isolated from 1 to 4-day-old Sprague-Dawley rats were treated with H/R injury according to the procedure in **Figure 2B**. Trypsin enzymic digestion and differential attachment methods were applied to separate and purify the primary cultures of neonatal ventricular myocytes. The cells at a final density of 1 × 10^5^/mL were cultured in a humidified incubator (95% air/5% CO_2_ at 37°C) in DMEM supplement with 10% fetal calf serum, streptomycin (100 μg/mL), penicillin (100 μg/mL) and 0.5 mM L-glutamine. Three days later, the cells were incubated with DMEM (low glucose) in an oxygen-free condition (95% N_2_/5% CO_2_) for 2 h (hypoxia). And then, the cells were incubated with DMEM-F12 in a well-oxygenated condition (95% air/5% CO_2_) for 2 h (reoxygenation).

The cells were randomly divided into 6 groups as follows (*n* = 8 per group): Control group, non-H/R cells cultured in DMEM medium; H/R group, cells treated with H/R procedure; Aspirin group, H/R cells preincubated with Aspirin (10 μM) for 24 h; 1, 10, 100 μM GC group, H/R cells preincubated with GC (1, 10, 100 μM) for 24 h.

### Reconstruction of CD40-Silencing Ventricular Myocytes

When ventricular myocytes reached 80–85% confluence, the cells in control group were transfected with pGPU6/Hygro while other groups’ cells were transfected with pGPU6/Hygro-CD40. The transfection should take 24 h using the GenePharma Transfection Reagent before the pretreatment of GC, Aspirin and H/R procedure.

### Analysis of Cell Vitality

Cell survival of ventricular myocytes was determined by MTT assay. After H/R procedure, 5 mg/mL MTT was added to the medium to incubate the cells for 4 h at 37°C. MTT was then removed, and cells were lysed with DMSO for 15 min. Absorbance was tested at wavelength of 490 nm by a microplate reader. Results were expressed as the percent of the optical density (OD) of control cells.

### Detections of TNF-α, IL-1β, and IL-6

Following 120 min reperfusion, all rats were deeply anesthetized, and blood samples were obtained. Cell supernatant was collected from medium after H/R procedure. The concentrations of TNF-α, IL-1β and IL-6 were detected by ELISA kits.

### Isolation of Cytoplasmic and Nuclear Protein

Cytoplasmic and nuclear proteins from cells were extracted by Nuclear-Cytosol Extraction Kit (Applygen Technologies Inc, Beijing, China). Cultured medium from plates was removed and astrocytes were detached with cold PBS and centrifuged at 1000 rpm for 5 min at 4°C. Pellets were resuspended in 200 μL of cytosol extraction buffer A and incubated on ice for 10 min. Then, 10 μL cytosol extraction buffer B was added, incubated on ice for 1 min and centrifuged at 1000 × *g* for 5 min at 4°C. The pellet contains crude nuclei. The supernatant was transferred to a new tube and further centrifuged at 12,000 × *g* for 5 min at 4°C. The supernatant is cytoplasmic proteins. The crude pellet was washed once with 100 μL cytosol extraction buffer A, spined for 5 min at 1000 × *g* and the supernatant was discard. Fifty microliter of cold nuclear extraction buffer was added and incubated on ice for 30 min. Finally, samples were centrifuged at 12,000 × *g* for 5 min at 4°C and supernatants were collected as nuclear proteins.

### Western Blot for ICAM-1, VCAM-1, iNOS, CD40, NF-κB p65, p-IκB-α, and IKK-β Expression in Ventricular Myocytes

The quantity of total protein was assessed by BCA assay. Fifty microgram of protein was loaded to SDS-PAGE gel and then, transferred to a PVDF membrane. After being blocked with 5% skim milk, the membrane was incubated with primary antibody (1:800) that recognized ICAM-1, VCAM-1, iNOS, CD40, NF-κB p65, p-IκB-α and IKK-β proteins. After incubation for 4 h at 37°C, horseradish peroxidase-conjugated secondary antibody (1:1000) was added to the membrane and the immune complexes was determined using an ECL plus kit. Images were taken using the Gel Imaging System with Quantity One software.

### Statistical Analysis

All data values were expressed as the mean ± standard deviation (SD). *Post hoc* tests were used to assess the statistical significance among means. Significance of difference between groups was determined by ANOVA followed by Bonferroni correction for multiple comparisons. Results were considered to be statistically significant when *P* < 0.05. All statistical figures were performed using Graph Pad Prism software (Version 5.0).

## Results

### GC Relieved the Outcomes of MI/R-Induced Inflammatory Injury

#### GC Reduced Infarct Size in MI/R Rats

**FIGURE 3:**
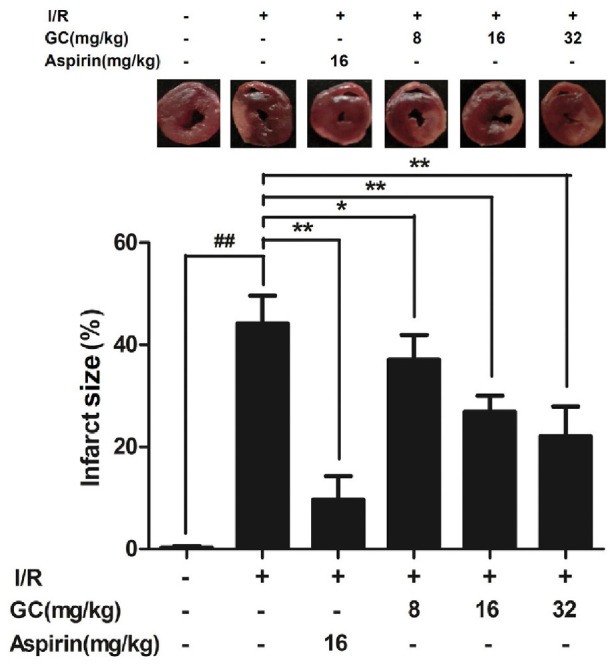
Effect of GC on infarct size in MI/R injury rat model. Treatment with Aspirin and GC significantly reduced the infarct size in MI/R injury rat model. Data were expressed as mean ± SD (*n* = 8). ^##^*P* < 0.01 vs. control group; ^∗^*P* < 0.05, ^∗∗^*P* < 0.01 vs. I/R group.

As shown in **Figure 3**, infarct size as a percentage of area at risk was 44.1 ± 5.5% (*P* < 0.01 vs. control group) in the I/R group, whereas administration with 8, 16, 32 mg/kg GC dose-dependently decreased infarct size to 37.1 ± 4.9% (*P* < 0.05), 26.9 ± 3.1% (*P* < 0.01), and 22.1 ± 5.8% (*P* < 0.01), respectively, compared with the I/R group. The infarct size of rats after administrated with 16 mg/kg Aspirin decreased to 9.7 ± 4.6% (*P* < 0.01) compared with I/R group. These findings strongly suggested that GC was effective at ameliorating MI/R injury.

#### GC Improved Cardiac Ultrastructural Characterization in MI/R Rats

**FIGURE 4:**
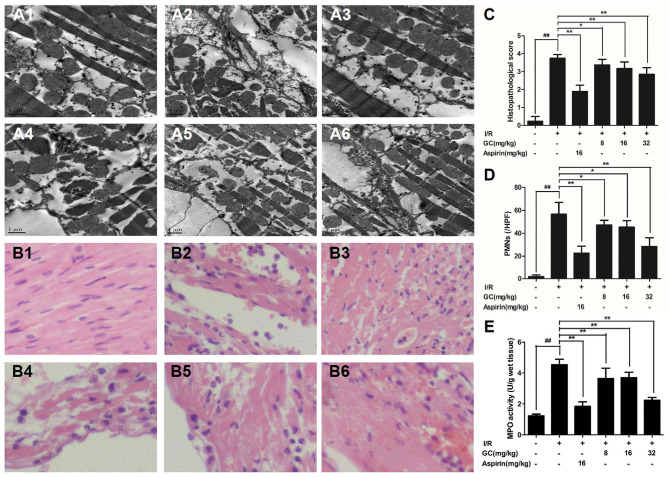
Effects of GC on the ultrastructure of myocardial tissue, histopathological changes, myocardial PMNs counting and MPO activity. **(A1–A6)** Representative transmission electron microscopy (TEM) observation of myocardial tissue injury for control group **(A1)**, I/R group **(A2)**, I/R + 16 mg/kg Aspirin group **(A3)**, I/R + 8 mg/kg GC group **(A4)**, I/R + 16 mg/kg GC group **(A5)**, I/R + 32 mg/kg GC group **(A6)**. **(B1–B6)** Representative light microscopic appearance of rat myocardial histopathological morphology (HE staining; original magnification × 200) for control group **(B1)**, I/R group **(B2)**, I/R + 16 mg/kg Aspirin group **(B3)**, I/R + 8 mg/kg GC group **(B4)**, I/R + 16 mg/kg GC group **(B5)**, I/R + 32 mg/kg GC group **(B6)**. **(C)** Effect of GC on histopathological scores, **(D)** effect of GC on myocardial PMNs counting and **(E)** effect of GC on MPO activity. The location of the histological images was taken in the infarcted area. Data were expressed as mean ± SD (*n* = 8). ^##^*P* < 0.01 vs. control group; ^∗^*P* < 0.05, ^∗∗^*P* < 0.01 vs. I/R group.

In the present study, mitochondrial alignment and myofibrillar banding appeared normal in the control group (**Figure 4A1**). However, in I/R group (**Figure 4A2**), mitochondria became swelling and degeneration, crista fragmentation and nuclear stained deeper. Z-band disalignment and myofibrillar degeneration occurred. There was no significant change of mitochondria and myofibrillar in Aspirin group (**Figure 4A3**). In 8 mg/kg GC group (**Figure 4A4**), damages on mitochondria and formation vacuoles alleviated. In 16 mg/kg GC group (**Figure 4A5**), mitochondria showed mild separation of cristae without swelling and vacuolation. In 32 mg/kg GC group (**Figure 4A6**), only a few mitochondria showed swelling and vacuolation.

#### GC Inhibited PMNs Infiltration in MI/R Rats

Pathological changes were applied to further evaluate the protective effect of GC on MI/R injury. After I/R occurred, the tissue in I/R injured area became necrotic, and the structure of intercalated disk and gap junction was severely impaired. However, treatment with GC and Aspirin could largely improve the histological features caused by I/R injury, characterized by alleviative inflammatory infiltration and recoverable structure of ischemic myocardial tissue (**Figures 4B1–B6**). As shown in **Figure 4C**, the histopathological scores were also decreased significantly by 8, 16, 32 mg/kg GC group compared with I/R group (*P* < 0.05, *P* < 0.01, *P* < 0.01). Above all, the total numbers of infiltrated and adherent PMNs in GC and Aspirin groups were remarkably less compared with that of the I/R group, as well as the histopathological score (**Figures 4C,D**).

To quantify neutrophilic infiltration, myocardial MPO activity was subsequently investigated. Very low MPO activity (1.21 ± 0.05 U/g protein) was tested in control group (**Figure 4E**). On the contrary, elevated MPO activity was found in I/R injured group, indicating that remarkable neutrophil accumulation appeared in the I/R area of the left ventricle (4.45 ± 0.23 U/g protein) (*P* < 0.01 vs. control group). However, compared with the I/R group, pretreatment with GC 8 mg/kg (4.20 ± 0.22 U/g protein, *P* < 0.05), 16 mg/kg (3.75 ± 0.30 U/g protein, *P* < 0.01), 32 mg/kg (2.22 ± 0.20 U/g protein, *P* < 0.01) and Aspirin 16 mg/kg (1.80 ± 0.25 U/g protein, *P* < 0.01) could prevent excessive MPO activity in myocardial tissue after I/R injury.

#### GC Inhibited Inflammatory Cytokines Overproduction in Serum of MI/R Rats

The concentration of inflammatory cytokines following I/R procedure in serum were detected by ELISA. **Table 1** showed that I/R injury elevated the levels of TNF-α by 2.98-fold (*P* < 0.01), IL-1β by 2.97-fold (*P* < 0.01), and IL-6 by 3.22-fold (*P* < 0.01), respectively, compared to control group. GC (8, 16, 32 mg/kg) dose-dependently decreased the levels of TNF-α by 17.2% (*P* < 0.05), 35.3% (*P* < 0.01) and 53.0% (*P* < 0.01), respectively, IL-1β by 21.9% (*P* < 0.01), 39.0% (*P* < 0.01) and 49.6% (*P* < 0.01), respectively, and IL-6 by 8.7% (*P* < 0.05), 29.6% (*P* < 0.01) and 44.1% (*P* < 0.01), respectively, compared with I/R group. In Aspirin group, the levels of TNF-α, IL-1β and IL-6 were decreased by 58.7% (*P* < 0.01), 60.0% (*P* < 0.01) and 57.4% (*P* < 0.01), respectively, compared with I/R group.

**Table 1:** Effects of GC on serum inflammatory cytokines after I/R in rats.

Group	Dose (mg/kg)	TNF-α (pg/mL)	IL-1β (pg/mL)	IL-6 (pg/mL)
Control		25.50 ± 6.91	86.63 ± 12.52	29.74 ± 5.07
I/R		76.10 ± 12.91^##^	257.23 ± 18.69^##^	95.87 ± 4.80^##^
I/R + Aspirin	16	31.41 ± 6.99^∗∗^	102.92 ± 7.31^∗∗^	40.87 ± 5.29^∗∗^
I/R + GC	8	63.01 ± 6.93^∗^	200.96 ± 8.71^∗∗^	87.55 ± 4.50^∗^
	16	49.23 ± 7.68^∗∗^	156.96 ± 13.88^∗∗^	67.53 ± 5.34^∗∗^
	32	35.79 ± 7.23^∗∗^	129.58 ± 7.05^∗∗^	53.58 ± 6.01^∗∗^

*Values were expressed as mean ± SD (*n* = 8). ^##^*P* < 0.01 vs. control group; ^∗^*P* < 0.05, ^∗∗^*P* < 0.01 vs. I/R group*.

#### GC Downregulated Overexpressions of ICAM-1, VCAM-1, and iNOS in Myocardial Tissue of MI/R Rats

**FIGURE 5:**
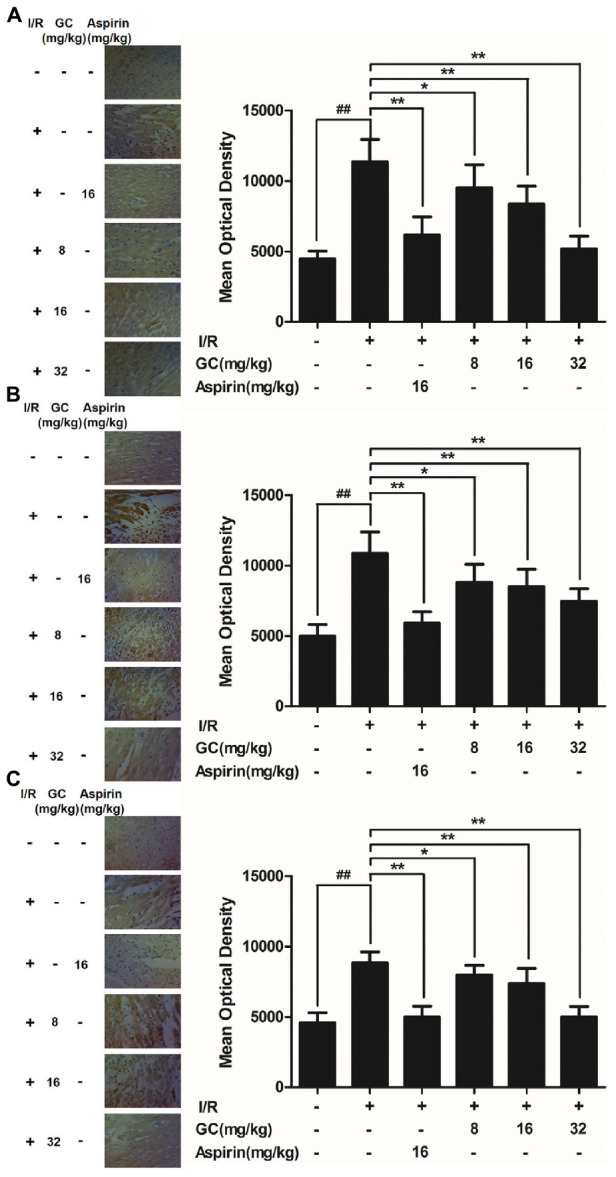
Effect of GC on ICAM-1, VCAM-1 and iNOS expressions in myocardial tissue after I/R procedure. The tissue was observed using a microscope at a magnification × 400. **(A)** GC decreased the expression of ICAM-1. **(B)** GC decreased the expression of VCAM-1. **(C)** GC decreased the expression of iNOS. There was a little expression of ICAM-1, VCAM-1 and iNOS in myocardial tissue of the control group. The expressions of ICAM-1, VCAM-1 and iNOS in I/R group were markedly increased. Administration of GC exhibited reduced expressions of ICAM-1, VCAM-1 and iNOS compared with the I/R group in a dose-dependent manner. Administration of Aspirin also significantly decreased the expressions of ICAM-1, VCAM-1 and iNOS compared with I/R group. The location of the histological images was taken in the infarcted area. Data were expressed as mean ± SD (*n* = 8). ^##^*P* < 0.01 vs. control group; ^∗^*P* < 0.05, ^∗∗^*P* < 0.01 vs. I/R group.

Immunostaining was used to visualize the expressions of ICAM-1, VCAM-1 and iNOS in myocardial tissue. Compared with the control group, I/R procedure significantly increased the expressions of ICAM-1, VCAM-1 and iNOS (**Figure 5**). However, the administration of Aspirin remarkably reduced the damage to myocardium and decreased the expressions of ICAM-1, VCAM-1 and iNOS compared with I/R group. Furthermore, pretreatment with 8, 16, 32 mg/kg GC remarkably reduced the expressions of ICAM-1, VCAM-1 and iNOS in contrast with I/R group. These results indicated that GC could protect against I/R injury through inhibiting expressions of key inflammatory factors and thus inhibiting inflammation.

### GC Protected Ventricular Myocytes against H/R-Induced Inflammatory Injury

#### GC Protected against Cell Insult after H/R Procedure in Ventricular Myocytes

**FIGURE 6:**
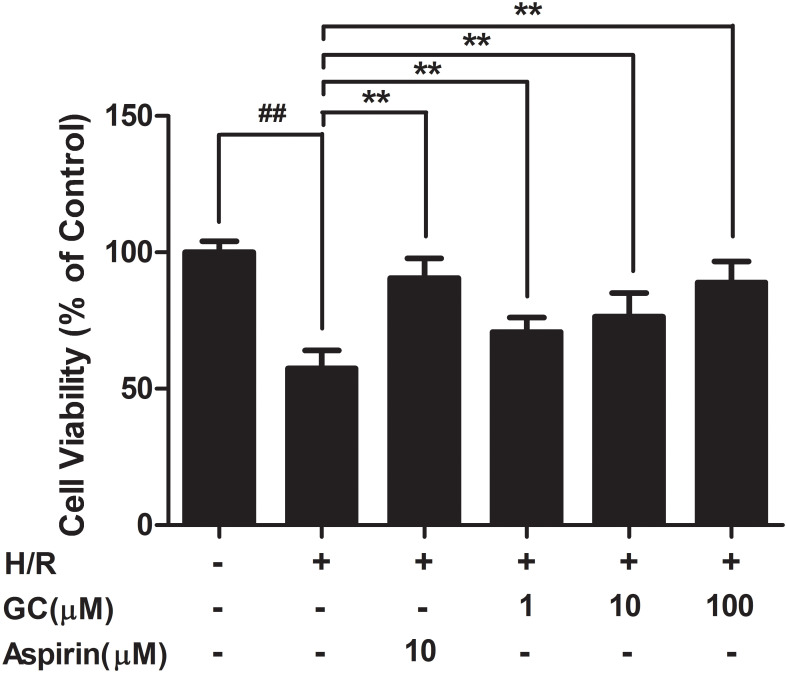
Effects of GC on cell viability after H/R procedure. Data were expressed as mean ± SD (*n* = 8). ^##^*P* < 0.01 vs. control group; ^∗^*P* < 0.05, ^∗∗^*P* < 0.01 vs. H/R group.

As shown in **Figure 6**, exposure of ventricular myocytes to H/R procedure resulted in a markedly reduce in cell viability (57.4 ± 5.0%, *P* < 0.01 vs. control group) as assessed by MTT assay. Pretreatment with 1, 10, 100 μM GC significantly increased the viability of ventricular myocytes in a concentration dependent manner (77.3 ± 6.9%, 83.5 ± 9.6%, 94.2 ± 5.0%, *P* < 0.01 vs. H/R group). Compared with H/R group, pretreatment with Aspirin also significantly increased the cell viability (93.3 ± 6.7%, *P* < 0.01).

#### GC Inhibited Inflammatory Cytokines Overreleasing after H/R Procedure in Ventricular Myocytes

As shown in **Table 2**, the production of TNF-α in H/R group were markedly increased by 8.47-fold (*P* < 0.01vs. control group). Pretreatment with 10 μM Aspirin and 1, 10, 100 μM GC could significantly reduce the levels of TNF-α by 68.3% (*P* < 0.01), 15.1% (*P* < 0.05), 22.4% (*P* < 0.01) and 71.0% (*P* < 0.01), respectively, compared with H/R group. After treatment with H/R procedure, the levels of IL-1β and IL-6 in H/R group remarkably increased by 26.69-fold (*P* < 0.01) and 12.51-fold (*P* < 0.01) compared with control group. Pretreatment with GC at the concentration of 1, 10, and 100 μM dose-dependently decreased the levels of IL-1β by 41.5% (*P* < 0.01), 52.9% (*P* < 0.01), and 69.6% (*P* < 0.01), respectively, and IL-6 by 6.1% (*P* < 0.01), 30.1% (*P* < 0.01), and 57.0% (*P* < 0.01), respectively, compared with H/R group. In addition, 10 μM Aspirin reduced the levels of IL-1β and IL-6 by 77.2% (*P* < 0.01) and 74.0% (*P* < 0.01) in contrast with H/R group.

**Table 2:** Effects of GC on the supernatant inflammatory cytokines of ventricular myocytes.

Group	Concentration (μM)	TNF-α (pg/mL)	IL-1β (pg/mL)	IL-6 (pg/mL)
Control		6.45 ± 1.41	34.02 ± 8.85	48.94 ± 6.82
H/R		54.61 ± 9.36^##^	907.96 ± 32.08^##^	612.16 ± 41.17^##^
H/R + Aspirin	10	17.33 ± 3.92^∗∗^	207.46 ± 81.12^∗∗^	159.03 ± 9.08^∗∗^
H/R + GC	1	46.38 ± 4.08^∗^	531.29 ± 54.70^∗∗^	574.98 ± 48.95^∗∗^
	10	42.35 ± 4.47^∗∗^	427.73 ± 43.31^∗∗^	427.75 ± 42.88^∗∗^
	100	15.86 ± 2.91^∗∗^	275.89 ± 78.23^∗∗^	263.16 ± 15.52^∗∗^

*Values were expressed as mean ± SD (*n* = 8). ^##^*P* < 0.01 vs. control group; ^∗^*P* < 0.05, ^∗∗^*P* < 0.01 vs. H/R group*.

#### GC Inhibited the Expression Level of CD40

**FIGURE 7:**
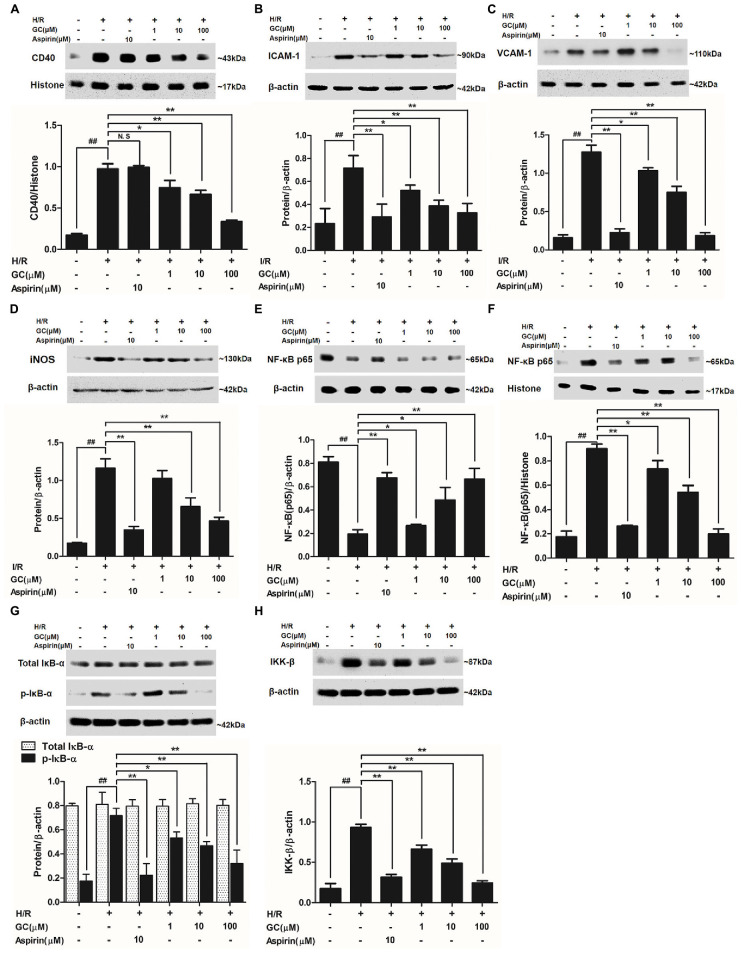
Effects of GC on the expressions of CD40, ICAM-1, VCAM-1, iNOS, NF-κB p65, p-IκB-α and IKK-β by Western blot after H/R procedure. **(A)** GC decreased the expression of CD40. **(B)** GC decreased the expression of ICAM-1. **(C)** GC decreased the expression of VCAM-1. **(D)** GC decreased the expression of iNOS. GC blocked the translocation of NF-κB p65 from cytosolic **(E)** to nuclear **(F)**. **(G)** GC down-regulated the expression of p-IκB-α. **(H)** GC decreased the expression of IKK-β. CD40, ICAM-1, VCAM-1, iNOS, p-IκB-α and IKK-β proteins were measured in cytosolic extract. The NF-κB p65 protein levels were assayed separately in cytosolic and nuclear extracts. Results were expressed as Protein/reference protein ratio. Data were expressed as mean ± SD of three independent experiments. ^##^*P* < 0.01 vs. control group; ^∗^*P* < 0.05, ^∗∗^*P* < 0.01 vs. H/R group.

As shown in **Figure 7A**, H/R group revealed apparently higher level of CD40 compared with control group. There was no significant difference between H/R group and Aspirin group in CD40 expression level. Nevertheless, 1, 10, 100 μM GC groups significantly decreased CD40 level in response to H/R injury (*P* < 0.05, *P* < 0.01, *P* < 0.01 vs. H/R group).

#### GC Inhibited Overexpressions of ICAM-1, VCAM-1, and iNOS after H/R Procedure in Ventricular Myocytes

As shown in **Figures 7B–D**, the expressions of ICAM-1, VCAM-1, and iNOS in ventricular myocytes significantly elevated to about 8.16-fold (*P* < 0.01), 3.06-fold (*P* < 0.01), and 6.74-fold (*P* < 0.01) after H/R procedure, compared with control group. While pretreated with GC (1, 10, 100 μM) exhibited reduced expressions of ICAM-1 by 19.1% (*P* < 0.05), 41.3% (*P* < 0.01) and 85.5% (*P* < 0.01), VCAM-1 by 27.2% (*P* < 0.05), 46.1% (*P* < 0.01) and 54.3% (*P* < 0.01) and iNOS by 11.8% (*P* > 0.05), 43.8% (*P* < 0.01) and 60.0% (*P* < 0.01) compared with H/R group in a concentration-dependent manner. In addition, 10 μM Aspirin reduced the levels of ICAM-1, VCAM-1 and iNOS by 82.4% (*P* < 0.01), 59.3% (*P* < 0.01) and 70.1% (*P* < 0.01), respectively, compared with H/R group.

#### GC Inhibited NF-κB p65 Translocation from Cytosol to Nucleus

Activation of NF-κB pathway is thought to be a key signaling event involved in the pathogenesis of MI/R. To determine whether GC inhibited H/R-induced cardiac NF-κB activation, we first examined the translocation of NF-κB p65 from cytoplasm to the nucleus by western blot. As shown in **Figures 7E,F**, a relatively high level of NF-κB p65 appeared in the cytoplasm of cells but low levels in nucleus in control group. Translocation of p65 from the cytosol into nucleus was evident in H/R group, whereas 10 μM Aspirin blocked this effect. As expected, such nuclear translocation was also concentration-dependently decreased by pretreatment of GC (1, 10, 100 μM). These results confirmed our hypothesis that GC was able to inhibit the translocation of p65 subunit to nucleus upon an inflammatory stimulus such as H/R injury.

#### GC Inhibited IκB-α Phosphorylation and IKK-β Activity

NF-κB translocation is preceded by the phosphorylation and ubiquitination of IκB-α, we then studied whether IκB-α was also in relation to the effect of GC on NF-κB pathway activation. First, we checked whether the total IκB-α had degradated. The results showed that the total IκB-α in each group was not different (**Figure 7G**). However, the level of p-IκB-α in H/R group remarkably increased by 4.08-fold (*P* < 0.01 vs. control group). In our study, we found a notably inhibitory effect on the H/R-induced IκB-α phosphorylation in the presence of Aspirin and GC. The results showed that Aspirin (10 μM) and GC (1, 10, 100 μM) all reduced the expressions of p-IκB-α by 68.9% (*P* < 0.01), 25.8% (*P* < 0.01), 34.7% (*P* < 0.01) and 55.2% (*P* < 0.01) compared with H/R group. These results indicated that GC could produce suppressive effect on NF-κB by disturbing the phosphorylation of IκB-α.

Substantial evidence unequivocally shows that a wide variety of influential factors regulate the activity of NF-κB, especially IKK-β. Then, we investigated the effect of GC on IKK-β activity. The results revealed a significant inhibitory effect on the H/R-induced IKK-β activation in the presence of GC (**Figure 7H**). The level of IKK-β in H/R group remarkably increased by 5.34-fold (*P* < 0.01 vs. control group). In contrast, pretreated with Aspirin (10 μM) and GC (1, 10, 100 μM) reduced the expressions of IKK-β (by 66.1, 28.9, 47.6, and 73.8%) (*P* < 0.01) compared with H/R group. These findings indicate that GC could mediate IKK-β activity.

### GC Failed to Alleviate H/R-Induced Ventricular Myocytes Inflammatory Injury in the Presence of CD40 Gene Silencing

#### GC Could Not Elevate Cell Viability in the Presence of CD40 Gene Silence

**FIGURE 8:**
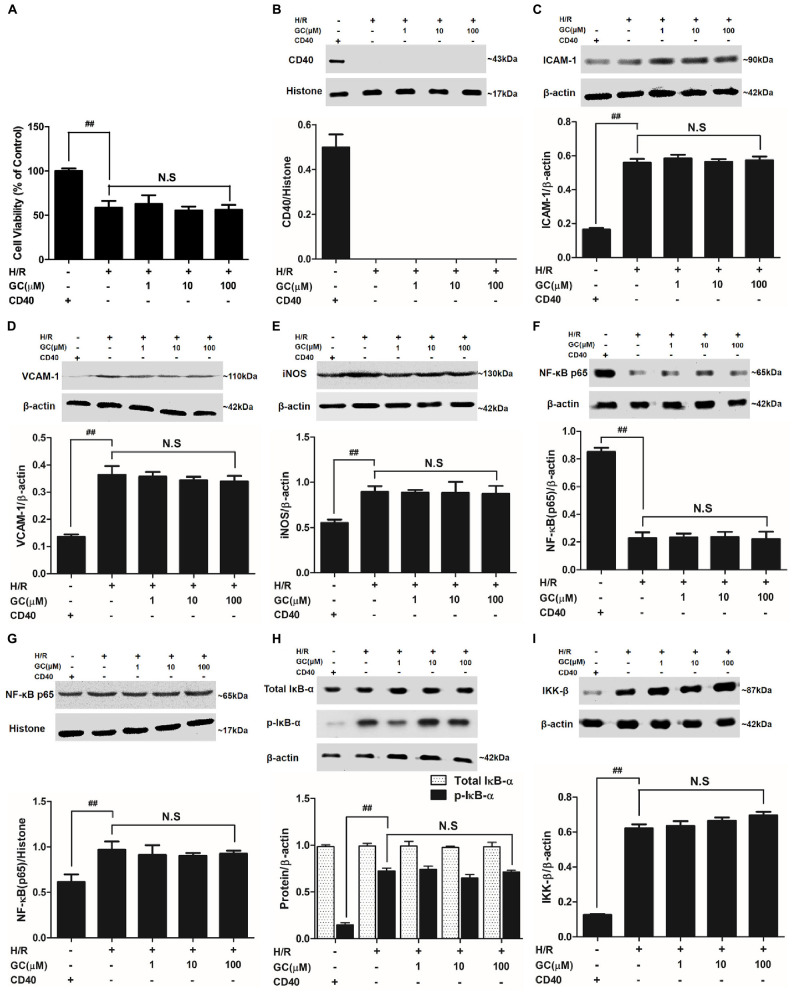
Effects of GC on cell viability **(A)** and the expressions of **(B)** CD40, **(C)** ICAM-1, **(D)** VCAM-1, **(E)** iNOS, **(F)** cytoplasm NF-κB p65, **(G)** nucleus NF-κB p65, **(H)** p-IκB-α and **(I)** IKK-β by Western blot after CD40 silencing procedure. Results were expressed as Protein/reference protein ratio. Data were expressed as mean ± SD of three independent experiments. ^##^*P* < 0.01 vs. control group; ^∗^*P* < 0.05, ^∗∗^*P* < 0.01 vs. H/R group.

The cell viability in H/R + CD40 silencing group was significantly lower than that in control group (*P* < 0.01). After the procedure of CD40 gene silence, GC could not increase the cell viability against to H/R injury (**Figure 8A**).

#### Successful and Stable CD40 Gene Silencing in Ventricular Myocytes

The ventricular myocytes preincubated with pGPU6/Hygro expressed little CD40 after transfection in control group, and preincubation of pGPU6/Hygro-CD40 successfully and stably inhibited CD40 expression in all groups (**Figure 8B**).

#### GC Could Not Inhibit Inflammatory Factors Expression in the Presence of CD40 Gene Silence

The expressions of TNF-α, IL-1β, IL-6, ICAM-1, VCAM-1 and iNOS in H/R + CD40 silencing group were significantly elevated compared with control group. After the procedure of CD40 gene silencing, GC could not regulate the expressions of TNF-α, IL-1β, IL-6, ICAM-1, VCAM-1 and iNOS (shown in **Figure 8** and **Table 3**).

**Table 3:** Effects of GC on the supernatant inflammatory cytokines of ventricular myocytes under the condition of CD40 gene silencing.

Group	Concentration (μM)	TNF-α (pg/mL)	IL-1β (pg/mL)	IL-6 (pg/mL)
Control		6.64 ± 0.94	35.40 ± 7.11	48.42 ± 9.09
H/R + CD40^-^		70.08 ± 11.39^##^	1036.01 ± 105.01^##^	603.53 ± 59.57^##^
H/R + GC + CD40^-^	1	69.27 ± 5.88	1041.32 ± 62.15	600.48 ± 107.81
	10	68.88 ± 7.47	1037.66 ± 52.72	601.44 ± 80.31
	100	68.56 ± 4.19	1023.08 ± 72.66	598.30 ± 60.98

*Values were expressed as mean ± SD (*n* = 8). ^##^*P* < 0.01 vs. control group*.

#### GC Had No Effect on NF-κB p65 Translocation, IκB-α Phosphorylation and IKK-β Activity in the Presence of CD40 Gene Silence

H/R + CD40 silencing group procedure significantly affected the translocation level of NF-κB, activation of IκB-α phosphorylation and up-regulation of IKK-β activity. Nonetheless, all GC groups showed no difference in NF-κB p65 translocation, IκB-α phosphorylation and IKK-β activity compared with H/R + CD40 silencing group (shown in **Figure 8**).

## Discussion

This is the first investigation studied on I/R rats subjected to GC to examine whether GC exerts significant protective effect against MI/R injury *in vivo*, whether CD40 is down-regulated by GC on ventricular myocytes in response to H/R injury *in vitro*, and whether NF-κB signal pathway plays a considerable role in the whole pathogenesis.

It has been broadly illuminated that inflammation played a vital role in the entire process of MI/R injury, and its effects refer to myocytes dysfunction, inflammatory cytokines overexpression, neutrophil accumulation and myocardium ischemia (Mohanta et al., 2014; Gragnano et al., 2017; Ma et al., 2017). Knowledge of the molecular mechanism underlying the proinflammatory processes of MI/R injury outlined above remains incomplete. However, information does exist regarding distinct signal pathways affected, including the activation of NF-κB signal pathway.

*Ginkgo biloba* is a unique tree species with no close living relatives and extracts of its leaves contain anti-inflammatory compounds including glycosides and terpenoids known as ginkgolides and bilobalides which have been reported to possess the properties of anti-cerebrovascular and anti-cardiovascular diseases (Ran et al., 2014). And, the different types of ginkgolides (A, B, C, J, and M) are the main pharmaceutical effective ingredients playing major part in the established medicinal functions of *G. biloba* extracts (Li et al., 2017b). Among them, the pharmacologic effects of GA and GB have been studied deeply now, especially focusing on their anti-inflammatory effects applied to treatment of vascular diseases (Yao et al., 2015; Anderson and Morrow, 2017; Li et al., 2017a). Nevertheless, GC which possesses the similarity chemical structure to GA and GB has not attracted the attention of us so far. Here, GC is investigated whether it possessed a strong anti-inflammatory property, which may be one of its molecular mechanisms of how GC exerts a protective effect in the pretreatment of MI/R injury.

It is clear that lack of blood to heart results in insufficient blood and oxygen transmitted to the beat of the heart muscle, named ischemia, following by damage or dysfunction of the cardiac tissue (Hansen, 1995). In addition, in this study we investigated the effect of GC in a rat model of MI/R injury in a prophylactic approach. Here, we uncovered that pretreatment with GC at 8, 16, 32 mg/kg per day for 7 days protected rat from I/R insult by occlusion of LAD coronary artery, through reduction of infarct size and improvement of myocardium damage. One of the most critical and accepted indicators reflecting therapeutic effect of MI/R injury is reduction of infarct size. In this study, our findings certified that GC was an efficient and promising target drug for prevention of MI/R injury via significant reduction of infarct size. GC also improves the ischemic myocytes damage and inhibits reperfusion-induced energy metabolism dysfunction, at least to a certain extent via suppression of myofibrillar degeneration and restoration of myocardial ultrastructure. Moreover, our recent research clearly provides additional and strong support that *G. biloba* has an irreplaceable role in the therapy of diseases as a valuable herb.

Importantly, neutrophils “shoot at first sight,” releasing reactive oxygen species and proteases, thereby causing extensive collateral damage to the ischemic myocardium and increasing infarct size (Stakos et al., 2015). Therefore, adherence of PMNs is identified as the beginning of a series of chain reaction after I/R injury. What is more, a large extent by PMNs infiltrating is a main inflammation response after MI/R. Activated PMNs will lead to a mass of inflammatory mediators’ release, which may directly result in the necrosis and apoptosis of myocytes (Hiroi et al., 2013; Xiao et al., 2017). Suppression of PMNs infiltration diminishes MI/R damage and potentially offers myocardial protection. In the present study, we found a significant aggravation of histopathological damage in *in vivo* model, indicating that there was a definite relationship between PMNs infiltration and MI/R injury. Whereas, administration with GC to rats could remarkably alleviate this situation that leukocyte infiltration was effectively attenuated, as determined by histopathological scores and the counting of PMNs. In addition, MPO activity which correlates closely with the number of neutrophils present in the heart was also evaluated. This result corresponds with respective histopathological scores data. Our investigation may provide a new insight: the potential use of GC as a cardio protective strategy to attenuate PMNs infiltration is clinically feasible for the prevention of MI/R injury.

Moreover, *in vitro*, we further applied H/R-treated ventricular myocytes to stimulate I/R injury *in vivo* thereby confirming the cardioprotective property of GC. Primary cultured neonatal ventricular myocytes stimulated by H/R were further applied to explore the anti-I/R injury property of GC *in vitro*. Increasing evidence suggests that inflammatory and immune responses have a profound impact on the damage process of myocytes directly exposed to H/R which had a strong influence on MI/R injury (Jin et al., 2017). Thus, based on the above reasons, H/R-induced ventricular myocytes *in vitro* model was applied to explore whether GC had protected cells from inflammatory damage caused by MI/R injury. In this study, we stated for the first time that GC increased cell viability after I/R-like insult, suggesting GC can elicit anti-MI/R injury effects by promoting tolerance and viability of cells injured by H/R-induced inflammation.

NF-κB could induce the transcription and expression of multiple cytokines related to immunity and inflammation and other relative gene so as to cause heart damage following pathologic process of I/R injury (Gao et al., 2016). However, there is some evidence that inhibition of NF-κB improves left ventricle (LV) remodeling and contributes to a decrease in cardiac dysfunction after MI/R injury (Lu et al., 2016). Moreover, an extensive body of research has demonstrated that inhibition of the indirect signal pathways mediated by NF-κB signaling can sharply suppress the inflammation after MI/R injury (Hayden and Ghosh, 2014; Xie et al., 2015). Furthermore, there is the certainty that phosphorylation of IκB-α acts as the trigger of NF-κB p50/p65 heterodimers’ translocation from cytoplasm to the nucleus (Mitchell and Vargas, 2016). Many studies have shown that aspirin treatment caused a strong decrease in NF-κB activation, inhibitor of IκB-α phosphorylation together with translocation of NF-κB p65 to nucleus and IKK-β activation (Shi et al., 2017). And many studies have shown that aspirin could improve I/R injury indexes as a positive and meaningful drug in the treatment of cardiovascular and cerebrovascular diseases (Basili et al., 2014). So we chose aspirin as the positive control to compare with the effect of GC in order to elucidate the exact mechanism. These conclusions are supported by our studies, where H/R group obviously elevated translocation of NF-κB p65, indicating an increased transcriptional activity of p65, whereas GC and aspirin effectively reversed this activated effect. In addition, stimulation with H/R procedure resulted in IκB-α phosphorylation and degradation, which was blocked by pretreatment with GC and aspirin. These results demonstrate that the molecular regulation of GC for I/R-induced inflammation involves in the inhibition of the NF-κB signal pathway, as shown by the reduction in phosphorylation of IκB-α and translocation of NF-κB p65.

It is now clear that NF-κB signaling is tightly regulated at the level of IκB phosphorylation. The IKK complex, composed of IKK (α, β, and γ), is activated by phosphorylation of IKK-α or IKK-β on serine residues within their activation loops either by upstream kinases or through autophosphorylation (Kondylis et al., 2017). The activated complex goes on to phosphorylate IκB-α, causing its ubiquitin-mediated degradation and release of the NF-κB subunits (Yang et al., 2017). And lots of evidence has shown that aspirin’s inhibitory effect of NF-κB has a close relationship with downregulation of IKK-β activity (Gao et al., 2014). Then, we detected whether GC has the similar impact like aspirin on IKK-β activity. Interestingly, IKK-β could also be inhibited by GC in H/R-induced intact ventricular myocytes. Therefore, we concluded that one of the anti-inflammatory targets of GC was IKK-β/NF-κB signaling.

Indeed, much evidence was given that activation of NF-κB occurred at the very early stages of I/R process and then sequentially modulated the occurrence of downstream inflammatory factors (Hostager, 2007). Therefore, we performed immunostaining and ELISA analyses to check whether GC could inhibit the inflammatory chain reaction caused by NF-κB-dependent gene transcription after I/R procedure. Our findings revealed that GC exerted its cardioprotective effects by an inhibitory mechanism that acted on excretion of pro-inflammatory mediators (TNF-α, IL-1β and IL-6) and expression of inflammatory proteins (ICAM-1, VCAM-1 and iNOS) through the NF-κB dependent pathway of inflammation. This is in accord with the investigation that studies the effects of GC on H/R-induced ventricular myocytes model *in vitro*. It means that, GC plays a protective role in the management of MI/R injury by blocking NF-κB pathway.

CD40 signaling is now widely accepted as a model of the non-canonical NF-κB pathway, which will strongly trigger its downstream signaling events (Jiang et al., 2011). More importantly, the expression of myocardial CD40 could significantly increase in the whole progression of inflammatory cardiovascular diseases (Hostager and Bishop, 2013). Therefore, CD40-mediated NF-κB activation is thought to be responsible for the massive inflammation and tissue damage after MI/R injury, and considered to be a valuable and promising therapeutic target against MI/R injury. Interestingly, we found that GC significantly down-regulated the expression of CD40 compared with H/R group.

According to the fact, we concluded that CD40 played the vital role in regulating inflammation for ventricular myocytes in response to H/R injury. Therefore, we silence the CD40 gene in ventricular myocytes stimulated by H/R to verify our hypothesis. As we expected, the cardioprotective effect of GC disappeared after CD40 gene was silenced. Consequently, it was evident that GC protected heart from MI/R injury through inhibiting NF-κB pathway via CD40. It is also consistent with other investigations reporting that inhibition of CD40 reduces the activation of NF-κB.

**FIGURE 9:**
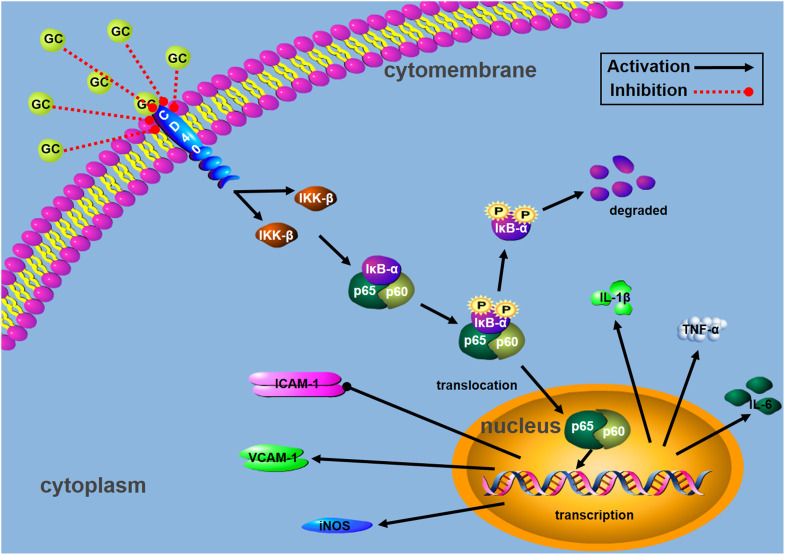
Schematic diagram describing the mechanism in the inhibitory effect of GC on H/R-induced ventricular myocytes inflammatory injury: 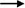 activation; 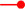 inhibition.

Taken together, our results obtained from this work demonstrated that GC protected rat from inflammatory insult by occlusion of LAD coronary artery and inhibited H/R-induced inflammatory damage to ventricular myocytes through blocking CD40 dependent NF-κB signal pathway on the basis of following evidence: (1) GC improved cardiac outcomes of MI/R injury and alleviated PMNs infiltration in rats, (2) GC inhibited overproduction of inflammatory factors (TNF-α, IL-1β and IL-6) and overexpression of inflammatory proteins (ICAM-1, VCAM-1 and iNOS) both *in vivo* and *in vitro*, (3) CD40 was down-regulated in ventricular myocytes in response to H/R injury, and (4) such effects were dependent on regulating CD40-NF-κB pathway (**Figure 9**).

## Conclusion

GC can exhibit significant cardioprotective effects through reducing infarct size, inhibiting inflammatory response, improving myocardial histological structure and alleviating PMNs infiltration during I/R injury. Inhibition of excessive inflammation via suppressing CD40/NF-κB signal pathway should be the key mechanism of GC in the protective of MI/R injury. Thus, GC will be a prospective and preventive agent in the management of MI/R injury.

## Ethics Statement

Animal experiments were carried out in accordance with the National Institutes of Health Guide for the Care and Use of Laboratory Animals. All procedures involved in the use of the laboratory animals were approved by the ethics committee of Shandong University (Permission No. 2013-092).

## Author Contributions

RZ, DH, ZL, YZ, and PL performed the research. JL, GY, SL, BH, and JbL designed the research study. RZ and CS analyzed the data. RZ wrote and edited the paper.

## Conflict of Interest Statement

The authors declare that the research was conducted in the absence of any commercial or financial relationships that could be construed as a potential conflict of interest.
