# Corrigendum: Ginkgolide C alleviates myocardial ischemia/reperfusion-induced inflammatory injury via inhibition of CD40-NF-κB pathway

**DOI:** 10.3389/fphar.2024.1492520

**Published:** 2025-01-06

**Authors:** Rui Zhang, Dan Han, Zhenyu Li, Chengwu Shen, Yahui Zhang, Jun Li, Genquan Yan, Shasha Li, Bo Hu, Jiangbing Li, Ping Liu

**Affiliations:** ^1^ Department of Pharmacy, Shandong Provincial Hospital Affiliated to Shandong University, Jinan, China; ^2^ Department of Pharmacy, Nanjing Drum Tower Hospital, The Affiliated Hospital of Nanjing University Medical School, Nanjing, China; ^3^ Minimally Invasive Urology Center, Shandong Provincial Hospital Affiliated to Shandong University, Jinan, China; ^4^ Department of Cardiology, Shandong Provincial Hospital Affiliated to Shandong University, Jinan, China

**Keywords:** ginkgolide C, myocardial ischemia/reperfusion injury, inflammation, CD40, NF-κB

In the published article, there was an error in Figure 3 as published. The heart slice picture of the I/R group in Figure 3 was incorrect as the authors used pictures stored in a folder with the wrong name. Therefore, when combining the pictures to create Figure 3, the wrong ones were used. The corrected Figure 3 and its caption appear below.

**FIGURE 3:**
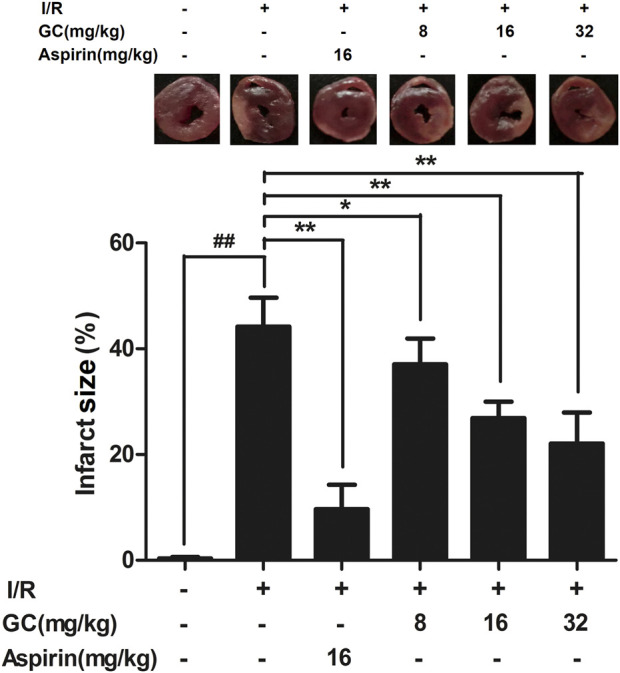
Effect of GC on infarct size in MI/R injury rat model. Treatment with Aspirin and GC significantly reduced the infarct size in MI/R injury rat model. Data were expressed as mean ± SD (n = 8). ^##^ *p* < 0.01 vs. control group; *p < 0.05, **p < 0.01 vs. I/R group.

In the published article, there was an error in Figure 4 as published. The pictures of Figure 4B were incorrect as the authors used pictures stored in a folder with the wrong name. Therefore, when combining the pictures to create Figure 4B, the wrong ones were used. The corrected Figure 4 and its caption appear below.

**FIGURE 4:**
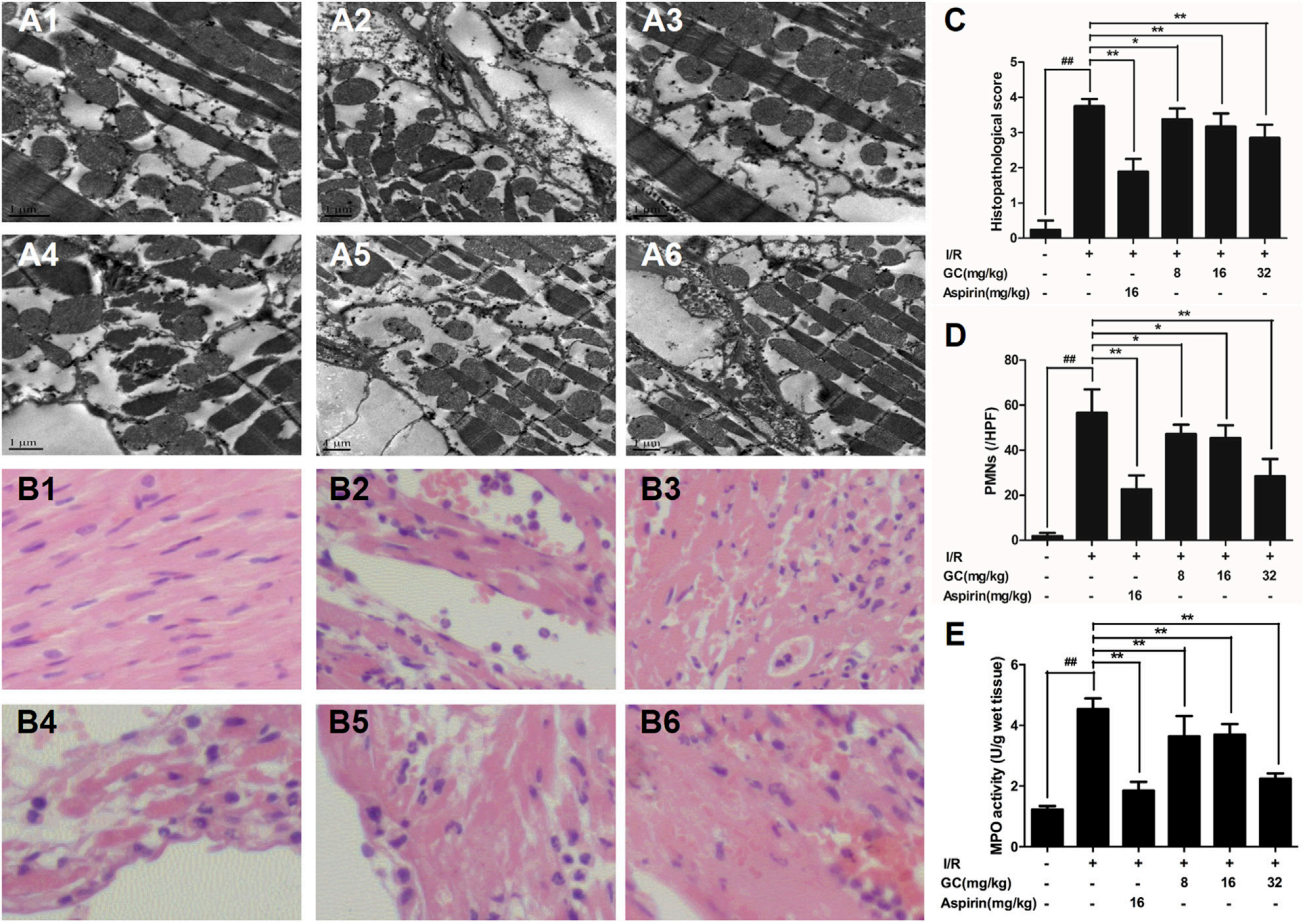
Effects of GC on the ultrastructure of myocardial tissue, histopathological changes, myocardial PMNs counting and MPO activity. **(A1–A6)** Representative transmission electron microscopy (TEM) observation of myocardial tissue injury for control group **(A1)**, I/R group **(A2)**, I/R + 16 mg/kg Aspirin group **(A3)**, I/R + 8 mg/kg GC group **(A4)**, I/R + 16 mg/kg GC group **(A5)**, I/R + 32 mg/kg GC group **(A6)**. **(B1–B6)** Representative light microscopic appearance of rat myocardial histopathological morphology (HE staining; original magnification × 200) for control group **(B1)**, I/R group **(B2)**, I/R + 16 mg/kg Aspirin group **(B3)**, I/R + 8 mg/kg GC group **(B4)**, I/R + 16 mg/kg GC group **(B5)**, I/R + 32 mg/kg GC group **(B6)**. **(C)** Effect of GC on histopathological scores, **(D)** effect of GC on myocardial PMNs counting and **(E)** effect of GC on MPO activity. The location of the histological images was taken in the infarcted area. Data were expressed as mean ± SD (n = 8). ^##^ *p* < 0.01 vs. control group; ∗*p* < 0.05, ∗∗*p* < 0.01 vs. I/R group.

In the published article, there was an error in Figure 5 as published. The pictures of Figures 5B, C were incorrect as the authors used pictures stored in a folder with the wrong name. Therefore, when combining the pictures to create Figures 5B, C, the wrong ones were used. The corrected Figure 5 and its caption appear below.

**FIGURE 5:**
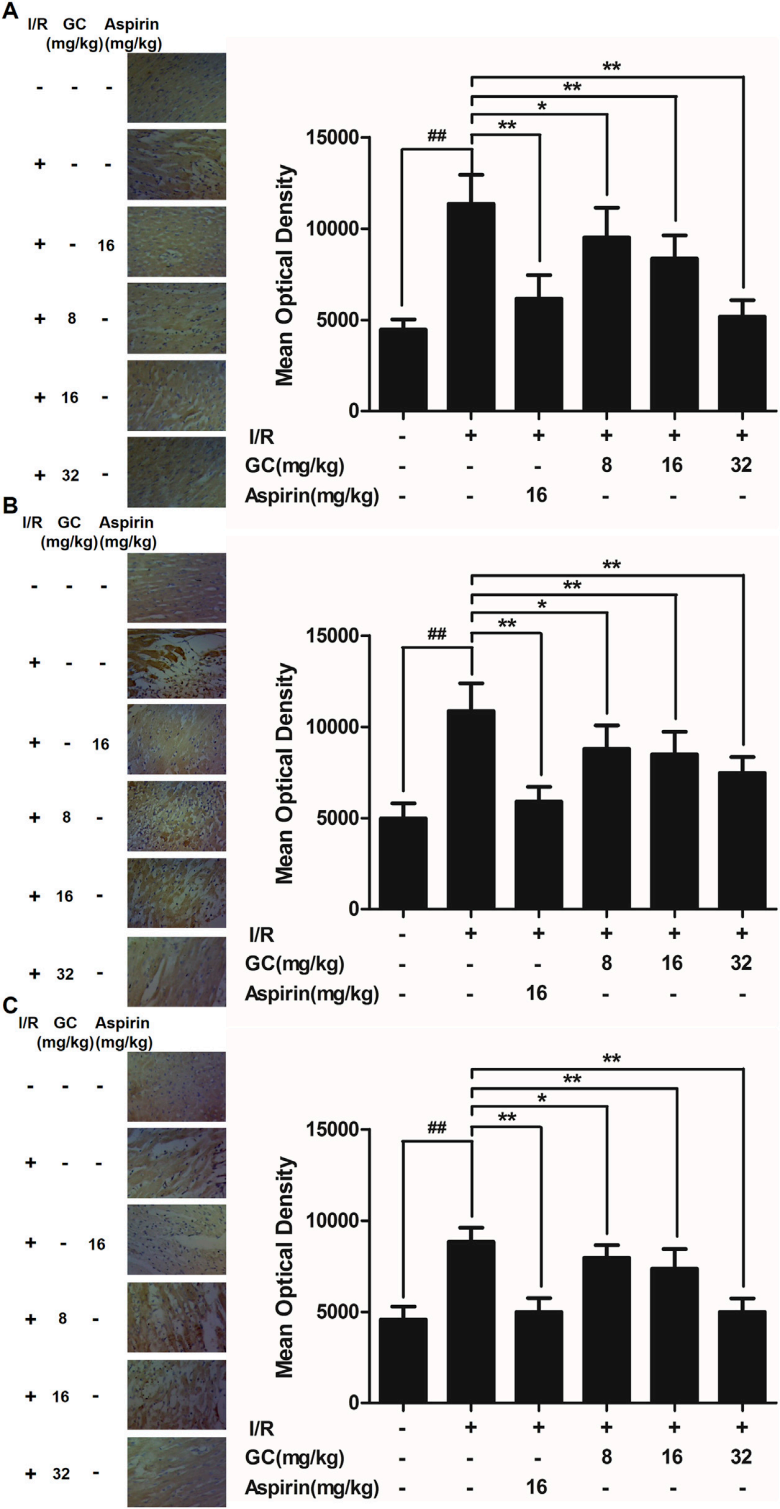
Effect of GC on ICAM-1, VCAM-1 and iNOS expressions in myocardial tissue after I/R procedure. The tissue was observed using a microscope at a magnification × 400. **(A)** GC decreased the expression of ICAM-1. **(B)** GC decreased the expression of VCAM-1. **(C)** GC decreased the expression of iNOS. There was a little expression of ICAM-1, VCAM-1 and iNOS in myocardial tissue of the control group. The expressions of ICAM-1, VCAM-1 and iNOS in I/R group were markedly increased. Administration of GC exhibited reduced expressions of ICAM-1, VCAM-1 and iNOS compared with the I/R group in a dose-dependent manner. Administration of Aspirin also significantly decreased the expressions of ICAM-1, VCAM-1 and iNOS compared with I/R group. The location of the histological images was taken in the infarcted area. Data were expressed as mean ± SD (n = 8). ^##^ *p* < 0.01 vs. control group; ∗*p* < 0.05, ∗∗*p* < 0.01 vs. I/R group.

In the published article, there was an error in Figure 7 as published. The target band VCAM-1 of Figure 7C was covered by the wrong band. Thus, the wrong protein band was displayed after combination. The corrected Figure 7 and its caption appear below.

**FIGURE 7:**
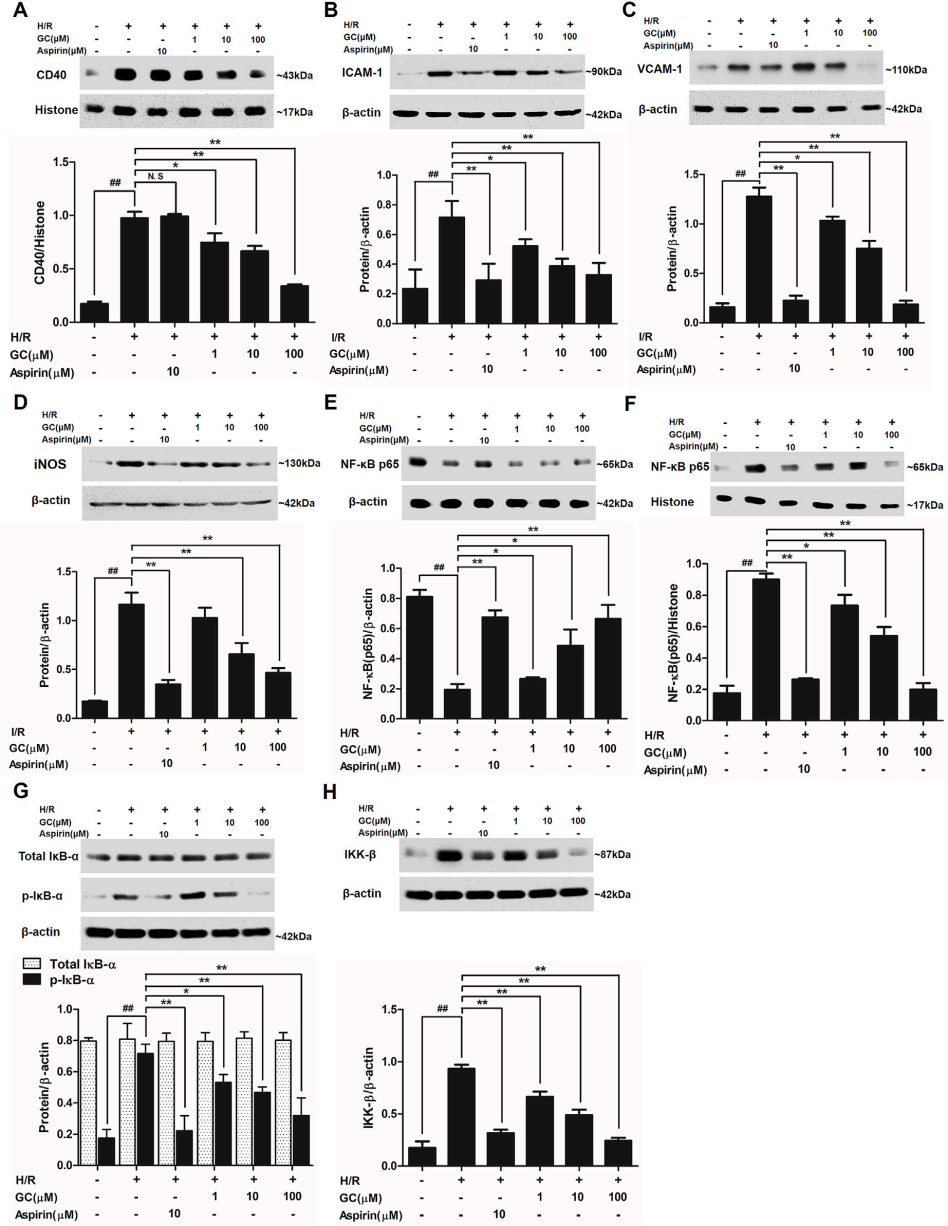
Effects of GC on the expressions of CD40, ICAM-1, VCAM-1, iNOS, NF-κB p65, p-IκB-α and IKK-β by Western blot after H/R procedure. **(A)** GC decreased the expression of CD40. **(B)** GC decreased the expression of ICAM-1. **(C)** GC decreased the expression of VCAM-1. **(D)** GC decreased the expression of iNOS. GC blocked the translocation of NF-κB p65 from cytosolic **(E)** to nuclear **(F)**. **(G)** GC downregulated the expression of p-IκB-α. **(H)** GC decreased the expression of IKK-β. CD40, ICAM-1, VCAM-1, iNOS, p-IκB-α and IKK-β proteins were measured in cytosolic extract. The NF-κB p65 protein levels were assayed separately in cytosolic and nuclear extracts. Results were expressed as Protein/reference protein ratio. Data were expressed as mean ± SD of three independent experiments. ^##^ *p* < 0.01 vs. control group; ∗*p* < 0.05, ∗∗*p* < 0.01 vs. H/R group.

In the published article, there was an error in Figure 8 as published. The target bands p-IκB-α of Figure 8H and β-actin of Figure 8E were covered by the wrong bands. Thus, the wrong protein band was displayed after combination. The corrected Figure 8 and its caption appear below.

**FIGURE 8:**
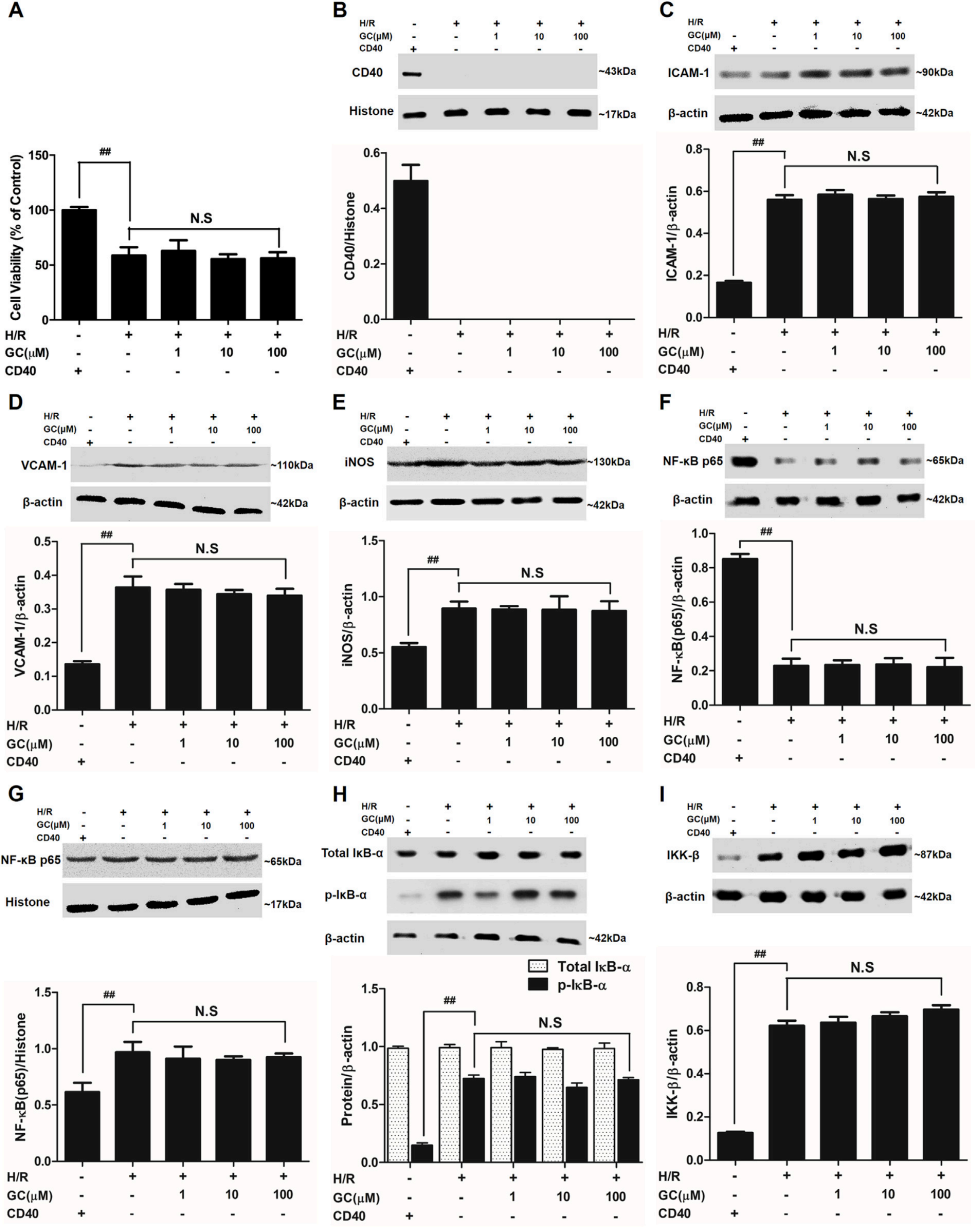
Effects of GC on cell viability **(A)** and the expressions of **(B)** CD40, **(C)** ICAM-1, **(D)** VCAM-1, **(E)** iNOS, **(F)** cytoplasm NF-κB p65, **(G)** nucleus NF-κB p65, **(H)** p-IκB-α and **(I)** IKK-β by Western blot after CD40 silencing procedure. Results were expressed as Protein/reference protein ratio. Data were expressed as mean ± SD of three independent experiments. ^##^ *p* < 0.01 vs. control group; ∗*p* < 0.05, ∗∗*p* < 0.01 vs. H/R group.

